# Attenuation of Toll-Like Receptor Expression and Function in Latent Tuberculosis by Coexistent Filarial Infection with Restoration Following Antifilarial Chemotherapy

**DOI:** 10.1371/journal.pntd.0000489

**Published:** 2009-07-28

**Authors:** Subash Babu, Sajid Q. Bhat, N. Pavan Kumar, R. Anuradha, Paul Kumaran, P. G. Gopi, C. Kolappan, V. Kumaraswami, Thomas B. Nutman

**Affiliations:** 1 National Institutes of Health—International Center for Excellence in Research, Chennai, India; 2 Science Applications International Corporation (SAIC)-Frederick, National Cancer Institute (NCI)–Frederick, Frederick, Maryland, United States of America; 3 Tuberculosis Research Center, Chennai, India; 4 Laboratory of Parasitic Diseases, National Institutes of Allergy and Infectious Diseases, National Institutes of Health, Bethesda, Maryland, United States of America; Leiden University Medical Center, Netherlands

## Abstract

*Mycobacterium tuberculosis* (Mtb) and filarial coinfection is highly prevalent, and the presence of filarial infections may regulate the Toll-like receptor (TLR)-dependent immune response needed to control Mtb infection. By analyzing the baseline and mycobacterial antigen–stimulated expression of TLR1, 2, 4, and 9 (in individuals with latent tuberculosis [TB] with or without filarial infection), we were able to demonstrate that filarial infection, coincident with Mtb, significantly diminishes both baseline and Mtb antigen-specific TLR2 and TLR9 expression. In addition, pro-inflammatory cytokine responses to TLR2 and 9 ligands are significantly diminished in filaria/TB-coinfected individuals. Definitive treatment of lymphatic filariasis significantly restores the pro-inflammatory cytokine responses in individuals with latent TB. Coincident filarial infection exerted a profound inhibitory effect on protective mycobacteria-specific TLR-mediated immune responses in latent tuberculosis and suggests a novel mechanism by which concomitant filarial infections predispose to the development of active tuberculosis in humans.

## Author Summary

Lymphatic filariasis afflicts over 120 million people worldwide, while *Mycobacterium tuberculosis* infects over 2 billion people worldwide. Almost 90% of infected people harbor latent tuberculosis infection with no clinical manifestations. Toll-like receptors (TLRs), pattern-recognition receptors that are present on host immune cells, constitute an important facet of host immune response initiation against a variety of pathogens. Because lymphatic filariasis and tuberculosis are co-endemic in many parts of the world and because TLR modulation is an important feature of both infections, we examined the effect of coexisting filarial infection on TLR-specific immune responses in latent tuberculosis by comparing cellular immune responses to Toll ligands in individuals with latent tuberculosis with or without concomitant lymphatic filariasis. We first examined modulation of TLRs at the gene expression level and determined that downregulation of TLR2 and 9 is associated with coexisting filarial infection. We then determined that Toll ligand-induced pro-inflammatory cytokine production—which is crucial in host defense against TB—is diminished in the presence of filarial coinfection. Treatment of filarial infection restores cytokine production in individuals with latent TB. Thus, we conclude that coexisting filarial infections might significantly compromise essential immune responses in tuberculosis and that treatment of filarial infections would potentially confer tremendous benefit in combating tuberculosis.

## Introduction

Lymphatic filariasis is a disease that afflicts over 120 million people worldwide. The parasites that cause the infection (*Wuchereria bancrofti*, *Brugia malayi*, and *Brugia timori*) are long lived and often induce asymptomatic (or subclinical) infections due, in large part, to the parasites' ability to manipulate the host immune system and to restrict inflammatory pathology [1]. Modulation of the host immune response involves a variety of strategies, including induction of regulatory networks and dysregulation of innate and adaptive immune responses [2]. Chronic filarial infections are associated with diminished expression and function of Toll-like receptors (TLRs) on antigen-presenting cells (APCs) and T cells [3],[4]. While the immune modulation associated with systemic filarial infections is primarily parasite-antigen specific, some bystander effects on routine vaccinations, allergic processes, and autoimmune diseases have been noted [5]. We have shown recently that filarial infection coincident with *Mycobacterium tuberculosis* (Mtb) infection significantly diminishes Mtb-specific Th1 (IL-12/IFN-γ) and Th17 (IL-23/IL-17) responses related to increased CTLA-4 and PD-1 expression (manuscript in press).

Mtb infects ∼2 billion people worldwide, with 90% of Mtb-infected individuals having latent infection. TLR signaling has been postulated to play an important role in the host resistance to Mtb [6],[7]. The control of tuberculosis (TB) requires clearly delineated Th1 responses (IL-12, IFN-γ, and TNF-α) and, to a lesser extent, Th17 responses (IL-17 and IL-23) [8]. Mtb and other mycobacteria contain well characterized TLR ligands that are potent in vitro stimuli of a number of proinflammatory cytokines including TNF-α and IL-12 [6]. A role for TLR signaling in host resistance to Mtb is further supported by the observation that mice deficient in MyD88, a major adaptor molecule required for signaling events by most TLR/IL-1R family members, show greatly enhanced susceptibility to aerosol infection with Mtb, equivalent to that observed with IFN-γ-deficient mice [9],[10],[11]. Infected MyD88^−/−^ animals, in addition to their loss of resistance to Mtb, display impaired proinflammatory cytokine synthesis, which was found to correlate with decreased nitric oxide synthase 2 expression and diminished IFN-γ synthesis [9],[10],[11]. In addition, TLR2 and 9 have been found to be essential in mediating immunity to Mtb [12].

Because filarial infections and TB are co-endemic in many parts of the world, we hypothesized that immune responses in latent TB could be modulated by diminished TLR expression and function induced by chronic, coexisting filarial infections. To this end, we examined the baseline and Mtb-specific expression of TLR1, 2, 4, and 9, as well as the induction of pro-inflammatory cytokines by TLR ligands. We observed that the presence of patent filarial infection altered profoundly the TLR-mediated cytokine responses in individuals with coexisting latent Mtb, an immune modulation that is reversible following treatment of the filarial infection.

## Methods

### Study population

We studied a group of 9 patients who were tuberculin skin test positive (PPD^+^) but filarial infection^−^ (hereafter PPD^+^Fil^−^) and 9 who were PPD^+^ and filarial infection^+^ (hereafter PPD^+^Fil^+^) in Tamil Nadu, South India (Table 1). Filarial infection was diagnosed by the presence of circulating filarial antigen first by using the ICT filarial antigen test (Binax, Portland, ME, USA) and then confirmed by positivity in the Trop Bio Og4C3 ELISA (Trop Bio Pty. Ltd, Townsville, Queensland, Australia). All subjects had positive skin test reactivity to intradermal tuberculin (2 TU). A positive tuberculin skin test was defined as an induration at the site of inoculation of at least 12-mm diameter to account for the high prevalence of environmental mycobacteria. This was based on the fact that a rigorous multivariate analysis of 280,000 subjects over a 15-year follow-up had previously demonstrated that subjects with 0–11-mm tuberculin skin test reaction to 2 TU PPD-S comprised the predominantly uninfected group and subjects with 12-mm or greater tuberculin skin test reaction comprised the predominantly infected group in South India [13]. All subjects had normal chest radiographs. None of the subjects had pulmonary symptoms (cough, fever, chest pain, hemoptysis) or a positive sputum for Mtb by smear microscopy and culture. All individuals were examined as part of a clinical protocol (NCT 01-I-N261) approved by Institutional Review Boards of both the National Institute of Allergy and Infectious Diseases and the Tuberculosis Research Center, and informed written consent was obtained from all participants. Fil^+^ individuals were treated with diethylcarbamazine (300 mg) per day for 7 days and a single dose of albendazole (400 mg) administered on the first day. Post treatment assessment of immune responses was performed 1 year following treatment.

**Table 1:** Characteristics of the study population.

	PPD^+^Fil^−^ (n = 9)	PPD^+^Fil^+^ (n = 9)
Median age (range)	48 (30–66)	42 (25–55)
Gender M / F	4 / 5	6 / 3
Treatment	No	Yes
Pathology	None	None
Tuberculosis skin test reaction (TU)	>12 mm	>12 mm
ICT card test	All −	All +
*W. bancrofti* circulating antigen levels U/ml (median)	<32 (<32)	177–32768 (3320)
*W. bancrofti* circulating antigen levels U/ml (median) – post treatment	NA NA	107–32768 (1024)

The lower limit of the assay detection was 32 U/ml.

NA , not applicable.

### Isolation of peripheral blood mononuclear cells (PBMCs)

Heparinized blood was collected and PBMCs isolated by Ficoll diatrizoate gradient centrifugation (LSM; ICN Biomedicals, Aurora, OH, USA). Erythrocytes were lysed using ACK lysis buffer (Biosource International, Camarillo, CA, USA). Cells were then washed and cultured in RPMI-1640 (BioWhittaker, Walkersville, MD, USA), supplemented with 20 mM glutamine (BioWhittaker), 10% heat-inactivated FCS (Harlan Bioproducts for Science, Madison, WI, USA), and 50 µg/ml of gentamycin (Mediatech, Herndon, VA, USA).

### Antigens

Mycobacterial PPD (Statens Serum Institute, Copenhagen, Denmark), Mtb-culture filtrate protein (CFP, kind gift of Dr. P. Selvaraj, Tuberculosis Research Center, Chennai, India) and tetanus toxoid (TT) were used as the antigenic stimuli. The TLR ligands (Invivogen, San Diego, CA, USA) used were: TLR2 ligand, Pam3CysSerLys4 (hereafter Pam3Cys); TLR4 ligand, ultrapure LPS; and TLR9 ligand, CpG ODN M362 (hereafter ODN).

### In vitro culture

PBMCs were cultured with PPD (10 µg/ml) or Mtb CFP (10 µg/ml) or TT (10 µg/ml) in 24-well tissue culture plates (Corning, Corning, NY, USA) at concentrations of 5×10^6^/well. After 24 hours, RNA was isolated and examined for TLR gene expression. PBMCs were also cultured with Pam3Cys (10 µg/ml), ultrapure LPS (10 µg/ml), or ODN (5 µM). After 24 hours, culture supernatants were collected and analyzed for cytokines.

### ELISA

The levels of cytokines in the culture supernatants were measured using Bioplex multiplex cytokine assay system (Bio-Rad, Hercules, CA, USA). The cytokines analyzed were IFN-γ, TNF-α, IL-12p70, IL-6, and IL-1β.

### RNA preparation

PBMCs were lysed using the reagents of a commercial kit (QIAshredder; Qiagen, Valencia, CA, USA). Total RNA was extracted according to the manufacturer's protocol (RNeasy mini kit; Qiagen), and RNA was dissolved in 50 µl of RNase-free water.

### cDNA synthesis

RNA (1 µg) was used to generate cDNA using TaqMan reverse transcription reagents according to the manufacturer's protocol (Applied Biosystems, Inc., Fullerton, CA, USA). Briefly, random hexamers were used to prime RNA samples for reverse transcription using MultiScribe reverse transcriptase.

### Real-time RT-PCR

Real-time quantitative RT-PCR was performed in an ABI 7500 sequence detection system (Applied Biosystems) using TaqMan Assays-on-Demand reagents for TLR1, 2, 4, and 9 and an endogenous 18 s ribosomal RNA control. Relative transcripts were determined by the formula
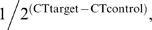
where CT is the threshold cycle during the exponential phase of amplification.

### Statistical analysis

Geometric mean was used as the measure of central tendency. Comparisons were made using either the Mann-Whitney U test (for unpaired data), the Wilcoxon signed rank test (for paired data), or Spearman rank correlation. All statistics were performed using GraphPad Prism version 5 for Windows.

## Results

### Filarial infection is associated with decreased baseline and Mtb-specific TLR2 and 9 expression in latent TB

**Figure 1:**
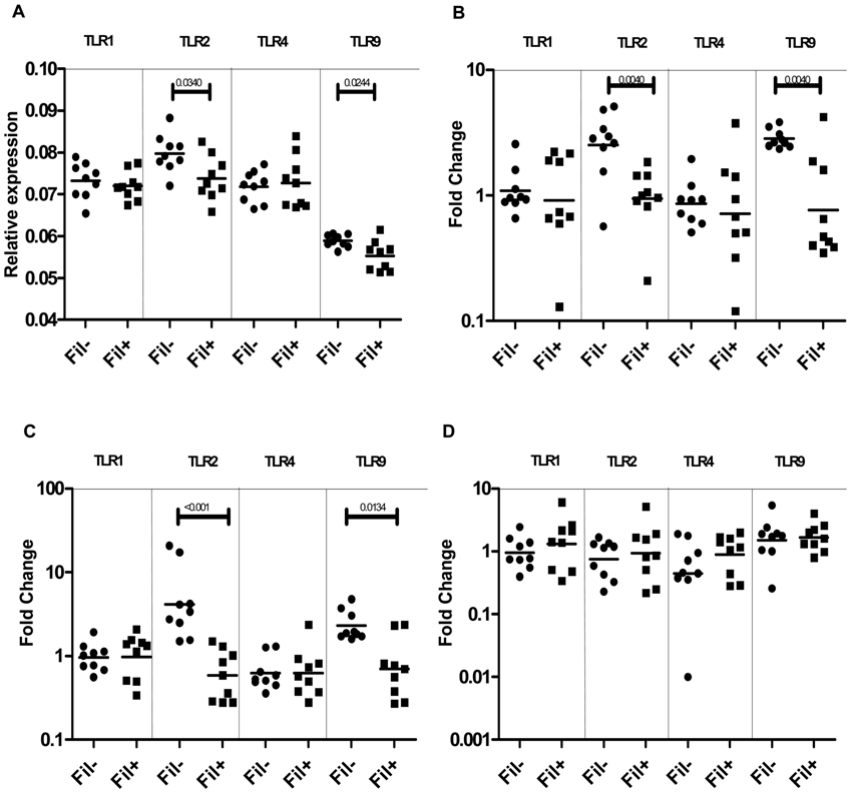
Filarial infection is associated with decreased expression of TLR2 and TLR9 at baseline and following PPD and CFP stimulation in latent TB patients. PBMC from PPD^+^Fil^−^ (*n* = 9) and PPD^+^Fil^+^ (*n* = 9) patients were examined at baseline (A) or stimulated with PPD (B) or Mtb CFP (C) or TT (D) for 24 hours, and TLR1, 2, 4, and 9 mRNA levels were measured by real-time RT-PCR. Results are shown as relative expression at baseline (A) or fold change over media control (B, C, and D). *p* values were calculated using the Mann-Whitney test.

To determine the impact of coexisting filarial infection on baseline and antigen-specific TLR expression of PPD^+^ individuals, and because filarial infection is associated with diminished expression of TLR1, 2, 4, and 9 [3], we examined the mRNA expression of these TLRs from PPD^+^Fil^−^ or PPD^+^Fil^+^ individuals ex vivo as well as following PBMC stimulation with PPD or culture-filtrate antigen from Mtb H37 Rv (Mtb CFP) or TT for 24 hours by RT-PCR. Both baseline (Figure 1A) as well as PPD- (Figure 1B) and Mtb CFP-specific (Figure 1C) induction of TLR2 and 9 was significantly lower in PPD^+^Fil^+^ patients (TLR2: geometric mean [GM] of 0.7373 in PPD^+^Fil^+^ vs. 0.7973 in PPD^+^Fil^−^ at baseline [P = 0.0340]; GM fold change of 0.9469 in PPD^+^Fil^+^ vs. 2.513 in PPD^+^Fil^−^ for PPD [P = 0.0040]; GM fold change of 0.5866 vs. 4.127 for CFP [P<0.0001] TLR9: GM of 0.5523 vs. 05889 at baseline [P = 0.0244]; GM fold change of 0.7660 vs. 2.834 for PPD [P = 0.0040]; GM fold change of 0.6989 vs. 2.297 for CFP [P = 0.0134]) compared with the PPD^+^Fil^−^ group. TT-specific TLR expression was examined as a control to TB antigens and did not exhibit any significant difference in expression (Figure 1D).

### Filarial infection is associated with diminished Pam3Cys and ODN - specific pro-inflammatory cytokine production in latent TB

**Figure 2:**
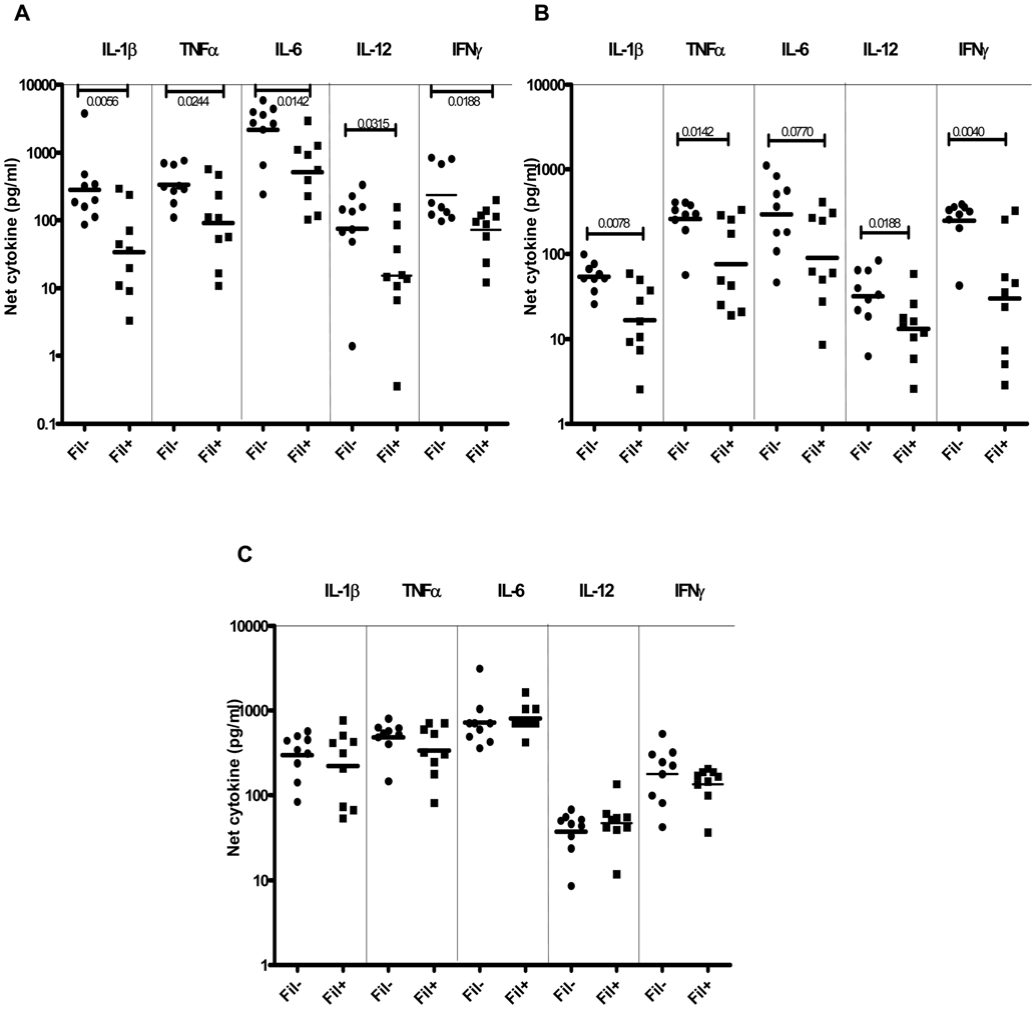
Filarial infection is associated with diminished cytokine responses to TLR2 and TLR9 ligands in latent TB patients. PBMC from PPD^+^Fil^−^ (*n* = 9) and PPD^+^Fil^+^ (*n* = 9) patients were stimulated with TLR2 ligand (Pam3Cys) (A) or TLR9 ligand (ODN) (B) or TLR4 ligand (LPS) (C) for 24 hours, and IL-1β, TNF-α, IL-6, IL-12p70, and IFN-γ cytokine levels were measured by ELISA. Results are shown as net cytokine production over media control. P values were calculated using the Mann-Whitney test.

To determine the impact of coexisting filarial infection on TLR-specific pro-inflammatory cytokine responses in PPD^+^ individuals, we stimulated PBMC from PPD^+^Fil^−^ or PPD^+^Fil^+^ with Pam3Cys (TLR2 ligand), ODN (TLR9 ligand), or LPS (TLR4 ligand) for 24 hours and measured the levels of IL-1β, TNF-α, IL-6, IL-12, and IFN-γ. As shown in Figure 2A and B, Pam3Cys and ODN induced significantly lower levels of IL-1β (GM of 33.60 pg/ml in PPD^+^Fil^+^ vs. 283.9 pg/ml in PPD^+^Fil^−^ for Pam3Cys [P = 0.0566]; GM of 16.61 pg/ml vs. 9.66 pg/ml for ODN [P = 0.0078]), TNF-α (GM of 91.19 pg/ml vs. 338.9 pg/ml for Pam3Cys [P = 0.0244]; GM of 76.99 pg/ml vs. 260.1 pg/ml for ODN [P = 0.0142]), IL-6 (GM of 511.1 pg/ml vs. 2157 pg/ml for Pam3Cys [P = 0.0142]; GM of 90.20 pg/ml vs. 295.6 pg/ml for ODN [P = 0.0770, not significant]), IL-12 (GM of 15.17 pg/ml vs. 21.37 pg/ml for Pam3Cys [P = 0.0315]; GM of 13.15 pg/ml vs. 32.12 pg/ml for ODN [P = 0.0188]) and IFN-γ (GM of 72.54 pg/ml vs. 237.1 pg/ml for Pam3Cys [P = 0.0188]; GM of 29.86 pg/ml vs. 294.4 pg/ml for ODN [P = 0.0040]) in the PPD^+^Fil^+^ compared with the PPD^+^Fil^−^ group. There were no significant differences in the production of the above-mentioned cytokines (Figure 2C) in response to LPS.

### Treatment of filarial infection is associated with enhanced Pam3Cys and ODN - specific pro-inflammatory cytokine production in latent TB

**Figure 3:**
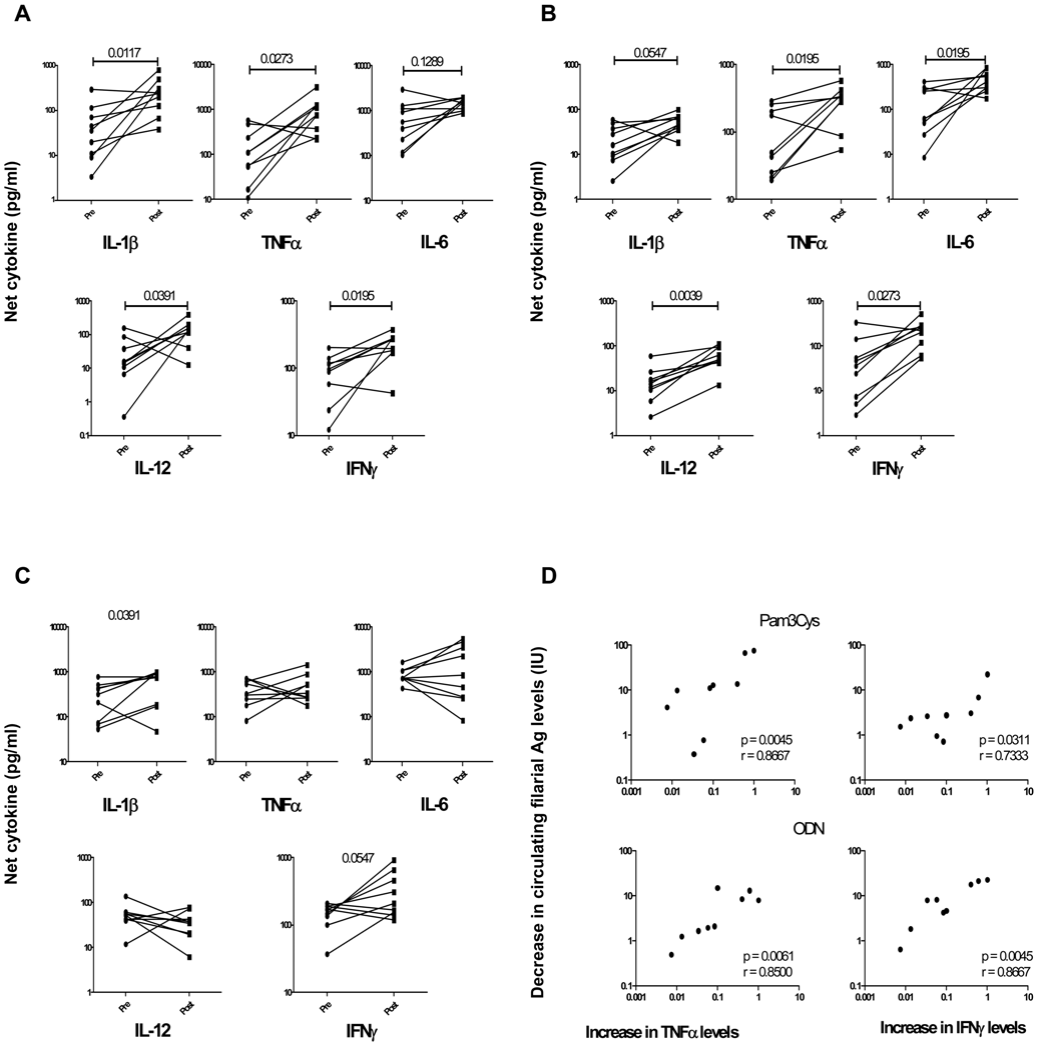
Treatment of filarial infection is associated with significantly enhanced cytokine responses to TLR2 and TLR9 ligands in latent TB patients. PBMC from PPD^+^Fil^+^ (*n* = 9) patients, pre and post treatment, were stimulated with ligands for TLR2 (Pam3Cys) (A), TLR9 (ODN) (B), or TLR4 (LPS) (C) for 24 hours, and IL-1β, TNF-α, IL-6, IL-12p70, and IFN-γ cytokine levels were measured. Results are shown as net cytokine production over media control. P values were calculated using the Wilcoxon signed rank test. (D) The correlation between the decrease in circulating filarial antigen levels and the increase in TNF-α and IFN-γ levels in patients with both lymphatic filariasis and latent TB following antifilarial treatment. P values were calculated using the Spearman rank test.

To determine whether antifilarial treatment could reverse the attenuated TLR-specific pro-inflammatory cytokine responses in PPD^+^ individuals with concomitant lymphatic filariasis, we stimulated PBMCs from PPD^+^Fil^+^, pre- and 1 year post-treatment, with Pam3Cys, ODN, or LPS for 24 hours and measured the levels of IL-1β, TNF-α, IL-6, IL-12, and IFN-γ. As shown in Figure 3A and B (and in comparison to pretreatment responses), Pam3Cys and ODN induced significantly increased production of IL-1β (GM of 31 pg/ml in pretreatment vs. 198.3 pg/ml post treatment for Pam3Cys [P = 0.0117]; GM of 16.61 pg/ml vs. 47.83 pg/ml for ODN [P = 0.0547, not significant]), TNF-α (GM of 91.19 pg/ml vs. 728.2 pg/ml for Pam3Cys [P = 0.0273]; GM of 72.73 pg/ml vs. 247.4 pg/ml for ODN [P = 0.0195]), IL-6 (GM of 511.1 pg/ml vs. 1396 pg/ml for Pam3Cys [P = 0.1289, not significant]; GM of 90.20 pg/ml vs. 418.1 pg/ml for ODN [P = 0.0195]), IL-12 (GM of 15.17 pg/ml vs. 105.4 pg/ml for Pam3Cys [P = 0.0391]; GM of 13.15 pg/ml vs. 53.46 pg/ml for ODN [P = 0.0390]) and IFN-γ (GM of 72.54 pg/ml vs. 201.4 pg/ml for Pam3Cys [P = 0.0195]; GM of 27.89 pg/ml vs. 174.6 pg/ml for ODN [P = 0.0273]) in the PPD^+^Fil^+^ group. Filarial infection treatment had minimal impact on LPS-induced production of the above-mentioned cytokines except IL-1β (P = 0.0391), which also showed an increase following antifilarial treatment (Figure 3C). Finally, as shown in Figure 3D, there was a strong relationship between the decrease in the circulating filarial antigen levels, a surrogate marker of treatment efficacy, and the increase in TNF-α and IFN-γ levels in response to Pam3Cys (P = 0.0045 and r = 0.8667 for TNF-α, and P = 0.0311 and r = 0.7333 for IFN-γ) and to ODN (P = 0.0061 and r = 0.8500 for TNF-α, and P = 0.0045 and r = 0.8667 for IFN-γ) following antifilarial treatment.

## Discussion

Parasitic helminths have evolved mechanisms to overcome and evade host immune responses to thrive in immune-exposed locations such as lymphatics, bloodstream, and gastrointestinal tract [2]. Most helminth infections induce relatively little disease in spite of extraordinarily high loads of infection. This subversion of the host immune response is achieved through induction of multiple layers of immunoregulation [2]. The interactions of helminth parasites with APCs are known to involve TLRs [14], pattern-recognition receptors that form a key component of microbial detection and host defense and are important in the initiation of host immune responses [15]. TLRs are involved in recognition of a wide spectrum of pathogens by binding to pathogen-associated molecular patterns [16]. In addition, TLRs control multiple APC functions and activate signals critically involved in the initiation of adaptive immune responses [17]. Downregulation of TLR-mediated immune responses—through dampening TLR-mediated cell signaling or through diminished TLR expression—appears to be an important immune evasion mechanism in some bacterial pathogens as well as in helminth infections [14],[18]. Thus, children with schistosomiasis have diminished responses to TLR ligands compared with uninfected children in the same endemic area [19]. Similarly, individuals with filarial infection have diminished expression of APC- and T cell-specific-TLR1, 2, 4, and 9 as well as decreased pro-inflammatory cytokine responses to TLR2, 4, and 9 ligands [3],[4].

Because immune-mediated protection against Mtb is characterized by strong Mycobacterium-specific Th1 responses [20], it has been postulated that coincident infections with helminth parasites could modulate these immune responses by driving Th2 and/or regulatory T cells that induce antiinflammatory responses [5]. Indeed, we have previously shown that filarial infection coincident with Mtb significantly diminishes Mtb-specific Th1 (IL-12/IFN-γ) and Th17 (IL-23/IL-17) responses related to increased expression of CTLA-4 and PD-1 [21]. Others have also shown that the poor immunogenicity of bacillus Calmette-Guérin vaccination in helminth-infected populations is associated with elevated TGFβ production [22]. Because filarial infections and Mtb infections are highly co-endemic in many parts of the world and often coexist within the same host, we wanted to examine the effect of filaria-induced downmodulation of TLRs on host responses to Mtb. In addition, the systemically circulating microfilariae may sequester in lung capillaries when not in the bloodstream, allowing them to be localized to the anatomic compartment associated with Mtb infection. Activation of the innate immune system via a number of pattern-recognition receptors, including TLRs, is thought to be a prerequisite for driving a protective adaptive immune response to Mtb [6]. Several studies have shown that Mtb contains a variety of pathogen-associated molecular patterns that serve as ligands for TLRs [6]. Based on a variety of in vitro studies, it has been suggested that TLR2, 4, and 9 are critically involved in induction of a Th1 response following infection with Mtb [6] however, in vivo data are not as clear cut. While some studies have shown no role for individual TLRs in protection against Mtb [9],[23],[24], others have shown that TLR2 and 9 are crucial in host resistance to Mtb [12],[25],[26].

Our study reveals a new mechanism by which coexisting filarial infections can modulate immune responses to Mtb infections. Dually infected individuals exhibit a significant decrease in the baseline as well as Mtb antigen-induced expression of TLR2 and 9. While the baseline decrease in expression of TLR2 and 9 mRNA has been shown by us previously, the impact on antigen-driven TLR expression is striking. The diminished ability to upregulate TLR expression following exposure to Mtb antigens suggests that individuals with filarial infections would be impaired in their immune response to TB and be at significant risk to develop active disease. The examination of cytokine responses to Toll-ligands in PPD^+^Fil^+^ patients revealed interesting differences in comparison with PPD^+^Fil^−^ individuals. First, IFN-γ and IL-12 were significantly downregulated in PPD^+^Fil^+^ individuals, suggesting that the IL-12/INF-γ pathway in patients with coincident lymphatic filariasis and latent TB is compromised. This has important clinical relevance, in that it is well known that mutations in the IL-12-IFN-γ–Stat1 pathway can lead to disseminated TB and atypical mycobacterial infections in humans [27]; in addition, mice deficient in IL-12 and/or IFN-γ are more susceptible to Mtb infection than their WT controls [28]. IFN-γ is the central effector molecule in macrophage elimination of bacteria, in that it induces increases in reactive nitrogen and oxygen compounds responsible for bactericidal activity as well as being central in the induction of autophagy, a process recently documented to play a critical role in eliminating mycobacteria within dendritic cells and macrophages [29],[30],[31]. Second, TNF-α production is significantly impaired in PPD^+^Fil^+^ individuals compared to the PPD^+^Fil^−^ individuals. TNF-α is another cytokine that plays an important role in preventing development of active clinical disease in individuals with latent TB [32]. Treatment of autoimmune diseases with TNF-α antagonists results in reactivation of Mtb and development of clinical disease in these individuals [33]. Thus, compromised production of TNF-α in response to Toll ligands suggests another mechanism that predisposes individuals with filarial infection to develop active TB. Finally, PPD^+^Fil^+^ individuals also exhibit significantly decreased production of IL-1β and IL-6 in comparison to PPD^+^Fil^−^ individuals in response to TLR ligands. Because IL-1β and IL-6 are important proinflammatory cytokines necessary for recruitment of innate effector cells such as macrophages, polymorphonuclear neutrophils, and NK cells to the infectious foci, the lack of induction of these cytokines would result in compromised activation of an effective adaptive immune response to Mtb [6]. In addition, IL-1β has also been shown to be essential for protection against Mtb infection in mice [34]. Thus, diminished functional responses to TLR2 and 9 ligands could potentially disrupt multiple mechanistic pathways operational in the control of Mtb infection.

Interestingly, treatment of filarial infection with a regimen of DEC and albendazole, resulted in significant lowering of worm burdens at 1 year post treatment as evidenced by the decrease in circulating filarial antigen levels. This is accompanied by the restoration of TLR-mediated cytokine responses to Pam3Cys and ODN. Thus, the diminished pro-inflammatory cytokine production observed in PPD^+^Fil^+^ individuals is abrogated following treatment, suggesting that the associated filarial infections are the cause of lowered cytokine responses in latent TB individuals. Moreover, there is a direct correlation between the decrease in filarial antigen load following treatment and the quantitative restoration of the cytokine responses in these individuals. Our findings highlight a novel mechanism by which a systemic helminth infection can modulate the cytokine response to Mtb through its effects on TLRs.

Thus, alteration of TLR expression and function in filaria-infected individuals with latent TB can have major implications in the control of latent TB infection. In addition, these findings also have significant implications for vaccine efficacy in helminth-endemic countries. Vaccines requiring a pro-inflammatory cytokine response for efficacy and those involving TLR agonists as adjuvants may not function optimally in the presence of helminth coinfection. The reversal of TLR modulation upon treatment of filarial infection suggests that elimination of this helminth infection in endemic areas might have a profound effect in the control of TB infection.
